# Instrumented Cone Penetrometer for Dense Layer Characterization

**DOI:** 10.3390/s20205782

**Published:** 2020-10-13

**Authors:** Jong-Sub Lee, Yong-Hoon Byun

**Affiliations:** 1 School of Civil, Environmental and Architectural Engineering, Korea University, Seoul 02841, Korea; jongsub@korea.ac.kr; 2 School of Agricultural Civil & Bio-Industrial Engineering, Kyungpook National University, Daegu 41566, Korea

**Keywords:** cone penetrometer, dense layer, dynamic response, in situ test, subsurface characterization

## Abstract

Subsurface characterization is essential for a successful infrastructure design and construction. This paper demonstrates the use of an instrumented cone penetrometer (ICP) for a dense layer characterization at two sites. The ICP consists of a cone tip and rods equipped with an accelerometer and four strain gauges, which allow dynamic driving, in addition to quasi-static pushing of the cone. The force and velocity of the cone are measured using the ICP instrumentation and compared with the N value, dynamic cone penetration index, and static cone resistance. A strong correlation has been observed between the total cone resistance estimated from the ICP and the dynamic cone penetration index and static cone resistance. After the correction of the dynamic cone resistance effect, the static component of the total cone resistance can be used as an alternative to a static cone resistance. This novel approach of soil resistance estimation using the ICP may be useful for dense layer characterization.

## 1. Introduction

One of the most important stages needed to achieve successful infrastructure design and construction is subsurface characterization. In situ tests are widely employed in geotechnical site characterization and soil property determination. Specifically, for cohesionless soil and highly fractured rock, in situ testing is suitable for soil resistance estimation without the requirement of any undisturbed samples utilized in laboratory tests. Accordingly, various methods for in situ testing have been established, as described in the following paragraphs.

The standard penetration test (SPT) is conducted by dropping a hammer with a weight of 623 N from a height of 760 mm on top of an anvil connected to the rods. At the end of the rods, the impact of the hammer drives the split-barrel sampler into the subsoil. The N value, the so-called blow count, denotes the number of blows required for the 300 mm penetration. However, to avoid gross errors in soil resistance estimation, the N value must be corrected for various factors, such as rod length, overburden pressure, sampler type and condition, borehole diameter, and transferred energy [1,2]. Moreover, a blow count must be referred to the energy transferred to the rod head and corrected to the same energy level for which any method or correlation was previously established.

The cone penetration test (CPT) is conducted using an electric cone penetrometer, cylindrical rods, rig, and data acquisition system. The electric cone penetrometer, which is mounted with transducers in and behind the cone tip, is quasi-statically pushed into the subsoil at a standard penetration rate of 20 mm/s; however, other rates may be applied, depending on the soil type so that accurate results can be obtained [3,4,5,6]. The continuous profiles of static cone resistance, sleeve friction, and pore water pressure are obtained from the CPT. In recent decades, the advantages of the CPT have been acknowledged, such as its simplicity, repeatability, and accuracy [7,8]. Furthermore, reliable correlations between the CPT and other penetration tests have been observed [9,10]. Despite the usefulness of the large-sized CPT rig for penetration through the dense layers, difficulties may arise in conducting the CPT in dense layers of intermediate geomaterials, such as weathered rock, till, or soils containing considerable gravel content, due to the limited availability of large-sized CPT rigs.

The dynamic cone penetrometer (DCP), which is a portable device designed for application to a shallow depth, is utilized for the evaluation of the in situ strength of granular materials ranging from fine-grained subgrades to weakly cemented layers. The standard DCP test provides the blow count per the desired depth, and in this test, a series of correlations between the DCP index and other engineering parameters of sandy soils have been observed [11]. For a more accurate characterization, an instrumented DCP was developed and used to evaluate the soil strength at a shallow depth [12,13,14,15,16,17]. However, the application of standard and instrumented DCPs is limited to fine-grained materials owing to their smaller cone diameter compared with coarse-grained materials. Accordingly, modification of the cone penetrometers is required for the characterization of deep and dense layers of intermediate geomaterials.

The dynamic probing super heavy (DPSH) tests specified in EN ISO 22476-2 [18] are commonly used in geotechnical site investigations of deep and dense layers. The DPSH equipment consists of a cone with a diameter of 50.5 mm and an apex angle of 90°, a rod with a diameter of 32 mm, and a hammer with a weight of 623 N dropped from a height of 750 mm. Similarly, the Texas cone penetrometer (TCP) has been utilized for the determination of the bearing capacities for pile design in Texas and Oklahoma [19,20,21]. The TCP with a diameter of 76 mm is driven by dropping a hammer with a weight of 760 N at a height of 610 mm. Similar to the SPT, the N value recorded from the TCP test exhibits a correlation with the compressive strength of intermediate geomaterials. However, the measured N values can be influenced by the energy transferred to the rod head. Moreover, the TCP test has not been widely accepted. Especially in the dense layer where the driving of cone is difficult or refused, a number of blow counts should be obtained, which is time consuming. Therefore, instrumentation of a large-sized cone penetrometer can be a promising method for a prompt and quantitative dense layer characterization.

The Becker penetration test (BPT) has been employed for the characterization of gravelly soils [2,22,23]. The large-diameter Becker drill exhibiting a closed-ended tip has the advantage of applicability for large-sized particles; however, the shaft resistance mobilizes along the drill string as it is driven into the subsoil. Ghafghazi et al. [24] developed an instrumented BPT to estimate the energy transferred at the tip and then to correct the blow count. However, a large driving system and blow counts are required for the instrumented BPT to reach a certain penetration distance owing to its large diameter.

This study presents the development of a new cone penetrometer incorporated with a drilling rig exhibiting high availability for dense layer characterization. It also suggests the use of a new index of soil resistance obtained from the dynamic response of the cone tip. The paper first provides a detailed description of the instrumented cone penetrometer (ICP), including the design, measurement system, mechanical force calibration, and test procedure. Then, the test results obtained at two sites are presented, and several correlations of the soil resistance index newly suggested by using the ICP are then discussed.

## 2. Instrumented Cone Penetrometer

### 2.1. Design

**Figure 1:**
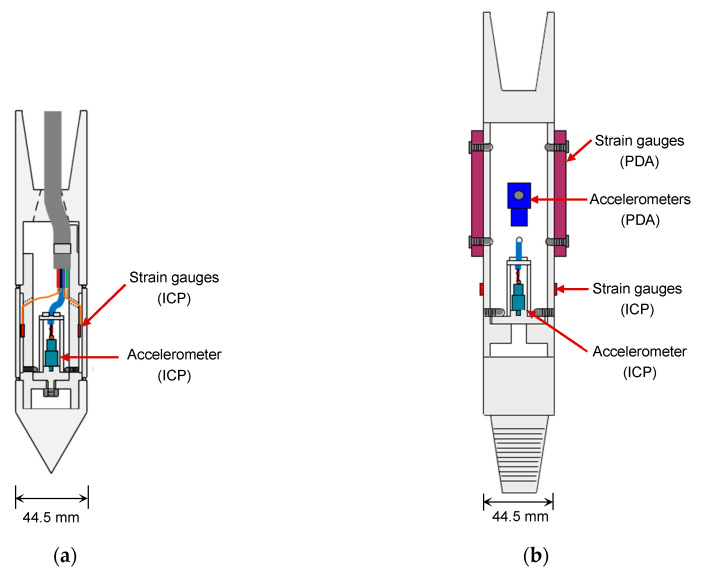
Schematic drawing of the instrumented cone penetrometer (ICP): (**a**) cone tip; (**b**) rod head. PDA—Pile Driving Analyzer.

The ICP consists of a cone tip, driving rod, and rod head. Figure 1 shows a schematic drawing of the cone tip and rod head equipped with electrical sensors. The cone tip and rod head have the same outside diameter of 44.5 mm as an AW-type rod. The cone tip has an apex angle of 60°, which is smaller than the cone apex angle (90°) of dynamic probing specified in EN ISO 22476-2 [18]. An accelerometer and four strain gauges were installed on the cone tip (Figure 1a). In the SPT, it was observed that the magnitude of acceleration above the sampler matched and even exceeded 10,000 g [25]. The accelerometer in the ICP can measure acceleration as large as 100,000 g in the frequency range of 1–10,000 Hz; it is rigidly mounted behind the cone tip. The acceleration measured at the cone tip can be applied for the calculation of velocity and displacement via integration or double integration. For force measurement, four strain gauges (350 Ω) were attached at the cone tip. The strain gauges were configured as a full bridge circuit and diametrically attached opposite to the surface of the inner rod in order to compensate for the effects of temperature change and eccentric loading [26,27,28]. When the force and velocity signals during a blow are known, the transferred energy can be calculated. The rod head also consists of an accelerometer and four strain gauges, which also allow for the evaluation of the energy transferred from the hammer to the rods (Figure 1b). For the verification of the instruments, the Pile Driving Analyzer (PDA) sensors, two strain gauges and two accelerometers, were also mounted on the rod head.

### 2.2. Data Acquisition System

The data acquisition system consists of a bridge box and a four-channel data logger, which were connected with the strain gauges and accelerometer, respectively. The data logger can be used to obtain data with a sampling rate of 96,000 Hz per channel simultaneously. During the static penetration, the sampling rate of the strain gauges used in force measurement is set to 10 Hz to measure the static cone resistance with high resolution. Conversely, for the dynamic response, the sampling rates of signals detected by strain gauges and accelerometers are set to 96,000 Hz. The dynamic response can be recorded during 310 ms, given that the number of data per blow is set to 30,000. In the data logger, the low-pass filter can be controlled up to a frequency of 20,000 Hz.

### 2.3. Calibration

**Figure 2:**
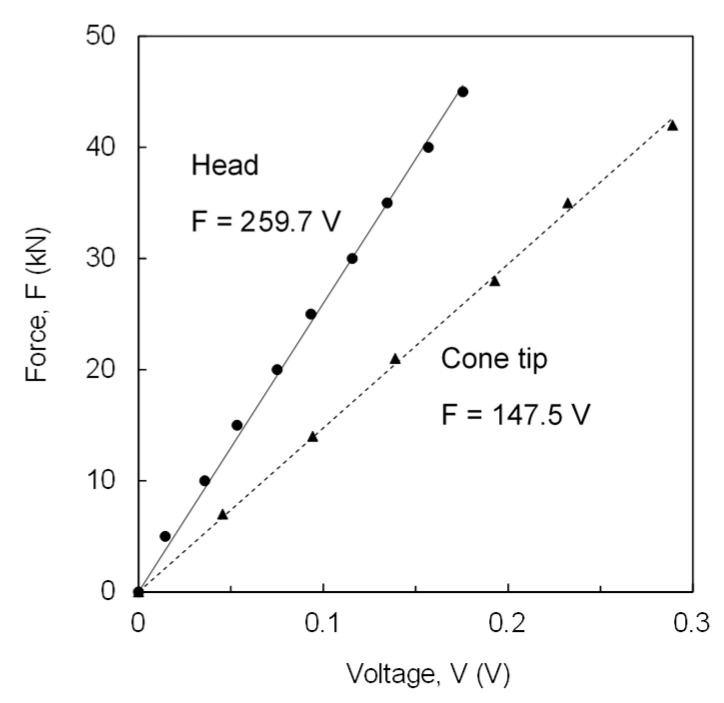
Mechanical force calibration of strain gauges. Input voltage is 5 V.

The strain gauges installed in the cone tip and rod head of the ICP are utilized for the mechanical force measurement. Using an axial loading frame with a maximum compression capacity of 1600 kN, calibrations of the strain gauges for the tip and the head were separately performed. The calibration results are presented in Figure 2. The slopes of the two force-voltage lines are the calibration factors for data acquisition.

### 2.4. Test Procedure

**Figure 3:**
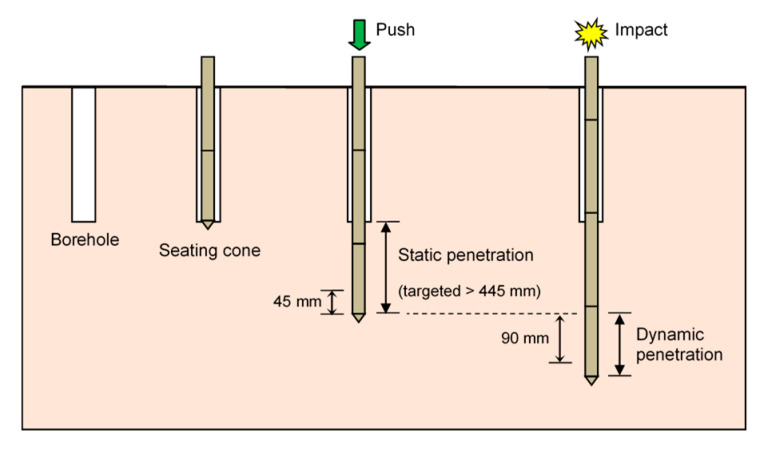
Procedure for a static–dynamic cone penetration tests using ICP at a depth.

A static–dynamic cone penetration test was conducted using the ICP, as shown in Figure 3. After drilling down the borehole to the desired depth, the ICP, which was connected to a typical SPT drilling rig with a weight of approximately 40 kN, was seated at the bottom of the borehole. Prior to static penetration, the ICP was allowed to hang freely to ensure a zero load and to account for temperature effects [29,30]. By using the drilling rig, the ICP was then pushed up to 600 mm into the subsoil to minimize the effect of borehole-drilling operations. Treen et al. [31] suggested that the penetration distance required for the effect of borehole drilling to become negligible varies from 5 to 10 times the cone diameter. In this study, the penetration distance of 445 mm corresponds to 10 times the cone diameter. Therefore, it is expected that the cone resistance measured in this manner approximates the cone resistance that would be measured during a regular CPT. By using the same drilling rig with a hammer having a weight of 623 N dropped from a height of 760 mm, the dynamic cone penetration was performed in sequence following the completion of the static penetration. In fact, the available distance for dynamic penetration depends on the type and depth of subsoils. The results of dynamic cone penetration tests using the ICP were obtained mostly within the first 90 mm penetration distance. Considering the similarity between the CPT and pile load test, the cone resistance could be influenced only by soil no more than twice the cone diameter below the cone tip [32]. It should be noted that the 90 mm distance corresponds to twice the cone diameters of the ICP.

**Figure 4:**
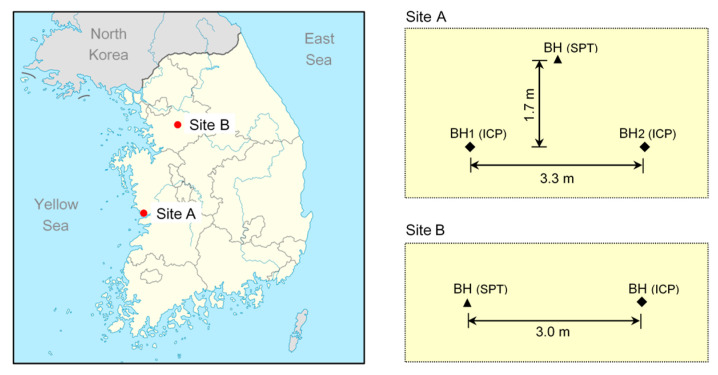
Location of sites and layout of the soundings in South Korea. BH—borehole.

Testing was conducted at two sites located on the southwest coast (site A) and the southern inland (site B) of the Korean peninsula, as shown in Figure 4. Two types of in situ tests were conducted: SPTs and static–dynamic cone penetration tests using the ICP at each site. For site A, the index tests were conducted on the disturbed samples obtained using the split-spoon sampler. Contrarily, the subsoils of site B included weathered rock and fill materials that contain considerable gravel content. Adequate samples were not obtained using the sampler due to the large-sized particles. The groundwater level for site A was 5 m, whereas that for site B was not detected up to the final depth of the tested boreholes. 

## 3. Results

### 3.1. Standard Penetration Tests

**Figure 5:**
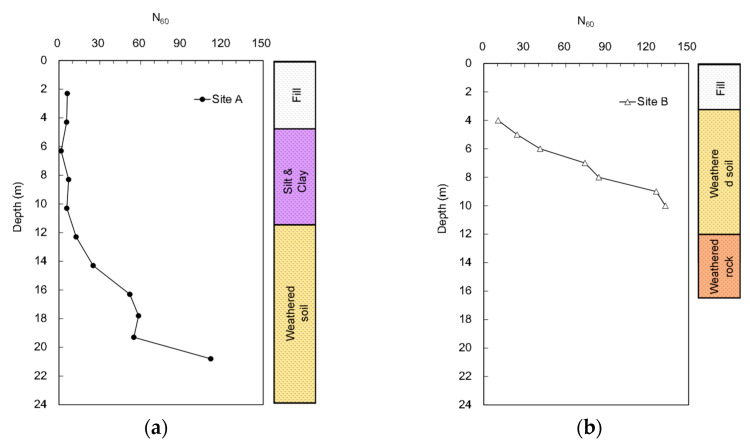
N value profiles measured from standard penetration test (SPT): (**a**) site A; (**b**) site B.

SPTs were conducted to obtain the blow count profiles for the two sites. Figure 5 presents the N value profiles for depths from 2.3 to 20.8 m at site A and for depths from 4 to 10 m at site B. In this study, the N values were corrected as follows:

(1) $$N_{60}=N\cdot \frac{\mathrm{ER}}{60\%}\cdot C_{R}$$

where ER denotes the energy ratio defined as the ratio of the transferred energy at the rod head to the theoretical potential energy of the hammer, and C_R_ denotes the factor of rod length. The transferred energy at the rod head was evaluated using the force–velocity (FV) method. The corrected blow count (N_60_) from the surface to the depth of 10.3 m at site A remains approximately unchanged under the 10 blows, increasing with depth from that point on. At a depth of 20.8 m, the soil is very dense, with a corrected N_60_ equal to 112. Considering site B, the N_60_ starts from 11 blows at a depth of 5 m and rapidly increases with depths up to 133 blows.

The disturbed soil specimens sampled during the SPTs at site A were used to determine the index properties of the soils, which are summarized as a function of depth in Table 1. The surface layer of site A was made up of fill materials down to a depth of 5 m. The soils obtained from a depth of 6.3 to 10.3 m were comprised of silt and clay (ML or CL), whereas those obtained from greater depths were classified as silty sand (SM).

### 3.2. Static Cone Penetration Tests

**Figure 6:**
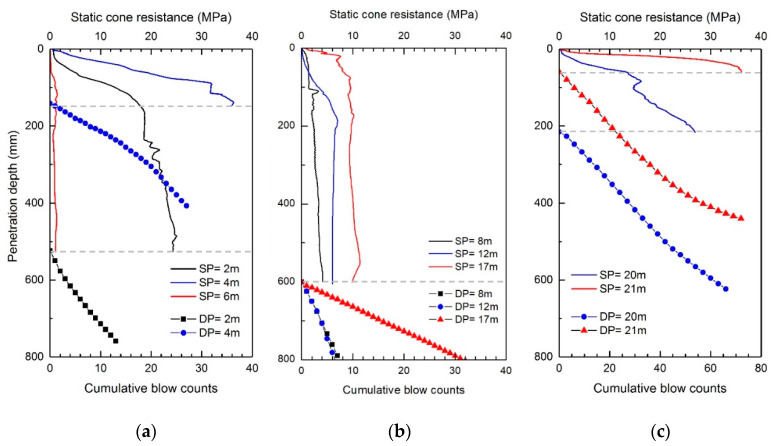
Profiles of static cone resistances and cumulative blow counts obtained from ICP in BH 1 at site A: (**a**) 2–6 m; (**b**) 8–17 m; (**c**) 20–21 m. SP and DP denote the static and dynamic cone penetration tests, respectively. Note that the data points of dynamic penetration at depths of 20 and 21 m indicate penetration depths at every three blows.

**Figure 7:**
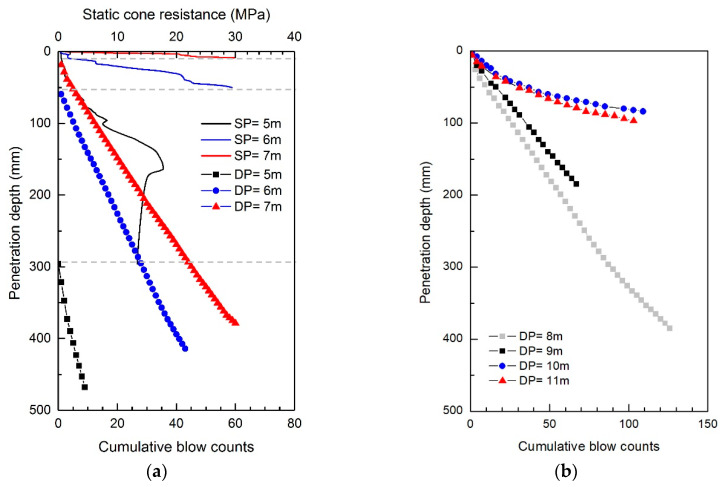
Profiles of static cone resistances and cumulative blow counts obtained from ICP at site B: (**a**) 5–7 m; (**b**) 8–11 m. SP and DP denote the static and dynamic cone penetration tests, respectively. Note that the data points at the depths of 8–11 m indicate penetration depths at every three blows.

The static CPTs were conducted using the ICP at sites A and B. At site A, two boreholes, BH1 and BH2, located at a 3.6 m spacing near the borehole for SPTs were utilized for the ICP application. The profiles of static cone resistance (q_s_) measured at each depth in BH1 are plotted in Figure 6. Figure 6 shows that the q_s_ at depths of 2 and 4 m rapidly increases over 20 MPa. It should be noted that at site A, the poorly graded sands with relatively large particles were laid down to a depth of 5 m. Below the depth of 6 m, the q_s_ hardly increases at depths ranging from 8 to 12 m below 10 MPa; however, the q_s_ starts to fluctuate at depths exceeding 14.5 m. Eventually, the q_s_ significantly increases as the depth of 21 m is approached; however, it was impossible for the generated reaction force to push the cone all the way to a penetration distance of 445 mm at depths of 4, 20, and 21 m. The q_s_ profiles at site B are plotted in Figure 7a. At site B, the static penetration tests were performed only at depths of 5 to 7 m, making it possible to push the cone only less than 445 mm penetration distances. For the depths of 6 and 7 m, where the q_s_ reached approximately 30 MPa, the cone could not penetrate into the subsoil due to the lack of reaction force. Considering the weight of the drilling rig employed in this study, the maximum reaction force corresponds to approximately 35 MPa.

**Figure 8:**
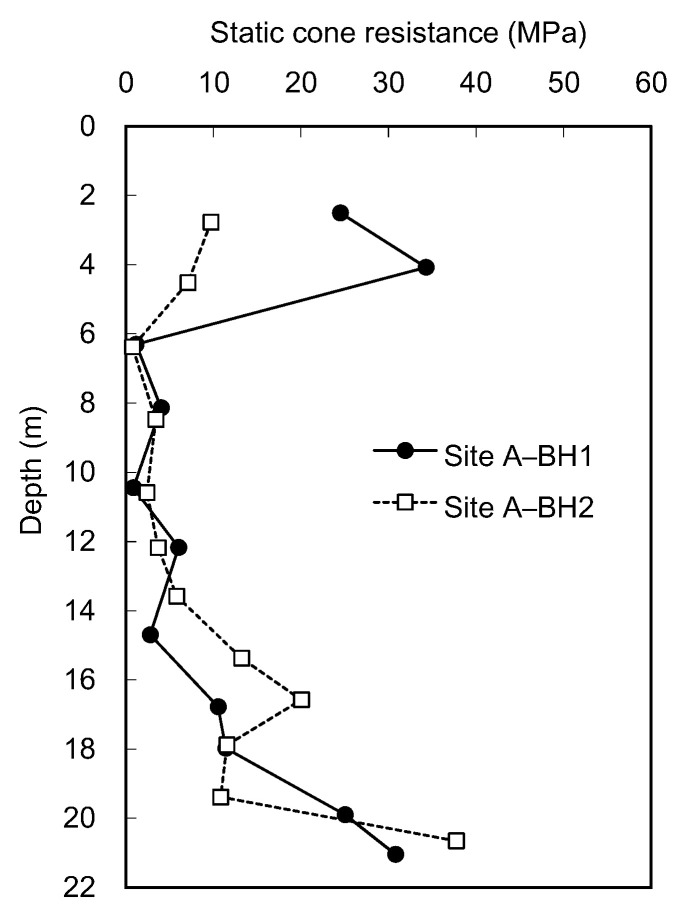
Profiles of average static cone resistances obtained from ICP.

The static cone resistances at each depth were averaged from the values measured for the last 45 mm penetration. The boring operation performed before the static cone penetration test at each depth may lead to stress relief of the borehole and reduce the q_s_ compared with the cone tip resistance (q_c_) obtained at the same depth in CPT. To minimize the effect of borehole-drilling operations on the measured q_s_ value, the values averaged for the last 45 mm penetration were applied. Note that the 45 mm penetration corresponds to 1 diameter of the cone above the cone tip at the final position. Considering that the equivalent average cone resistance is determined above and below 1 diameter of the pile tip [33], the q_s_ averaged for the last 45 mm penetration can be correlated with the results of dynamic cone penetration tests obtained below the final position of the cone tip after the completion of the static cone penetration test. The profile of the average q_s_ along the entire depth is plotted in Figure 8. At site A, the trends of average q_s_ obtained from BH1 and BH2 were similar, except for the depth of 4 m. The variance in q_s_ can be expected, as the sample at the 4 m depth includes particles larger than 19 mm. The q_s_ at site B was excluded due to the lack of reaction force. Note that at depths deeper than 20 m at site A, the required penetration distance of 445 mm was also not achieved to estimate the average q_s_.

### 3.3. Dynamic Cone Penetration Tests

The dynamic cone penetration tests were conducted after the completion of the static cone penetration test at each depth. During the driving of the ICP, the blow counts and penetration depth were recorded. Figure 6 presents the profiles of the cumulative number of blows obtained at site A. At the depths of 2 and 4 m, the cumulative blow count numbers corresponding to the first 100 mm penetration (BC_100_) are 5 and 14, respectively. At a 6 m depth, the dynamic cone penetration test was not applicable as the penetration distance per blow was greater than 450 mm. Note that the q_s_ is smaller than 1 MPa within a typical range of q_s_ in clay. From the depths of 8 to 12 m, the BC_100_ is approximately 4 blows, whereas at depths of 17 and 18 m, it is approximately 17 blows. At the depths of 20 and 21 m, the BC_100_ was 15 blows for both. Overall, the variation in the BC_100_ along the depth is similar to that in the q_s_, but not proportional to the q_s_. Note that the blow count is influenced by both the tip resistance and sleeve friction, whereas the q_s_ is influenced only by the tip resistance. The blow counts obtained at site B are plotted in Figure 7. For the entire depths, the profiles of site B exhibit larger blow counts compared with site A. The BC_100_ at a depth of 6 m is 11 blows and reaches 26 blows at a depth of 8 m. At the depths of 9–11 m, a slope change in the profiles of the cumulative blow counts occurs. The blow counts for the 100 mm penetration were 35 and 109 at the depths of 9 and 11 m, respectively.

**Figure 9:**
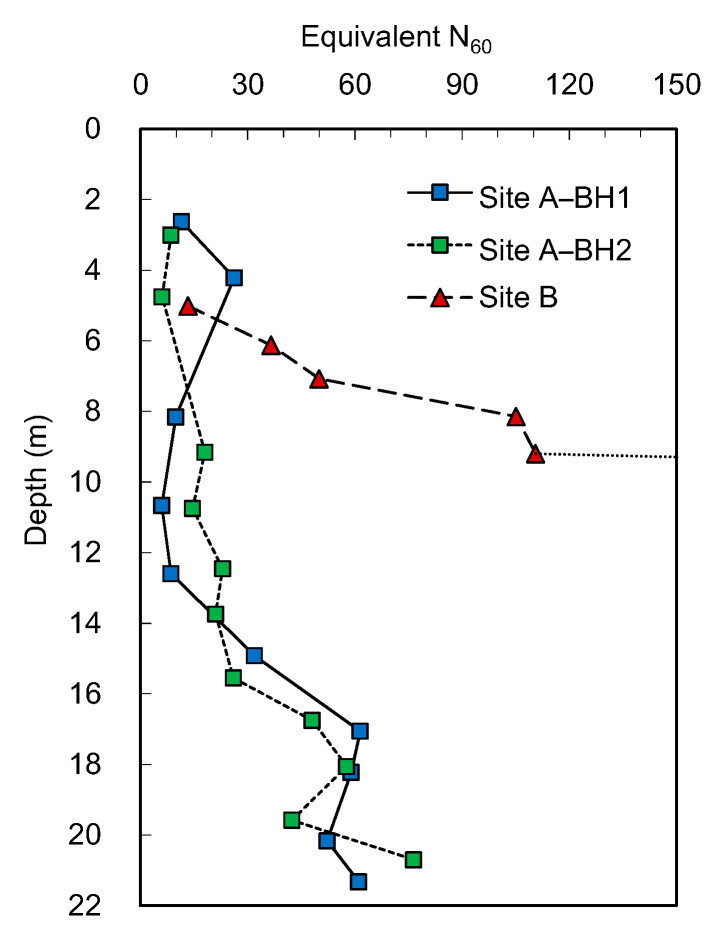
Profiles of the equivalent N_60_ obtained from ICP.

To obtain a blow count that would correspond to the same penetration length (300 mm) as the N_60_, the blow counts following the first 150 mm penetration distance are utilized. Note that the blow counts for the first 150 mm penetration are discarded in SPT. In the case that the dynamic cone penetration tests were performed below the 450 mm penetration, the blow count can be multiplied by a conversion factor inversely proportional to the penetration length, i.e., a conversion factor of 3 for the 100 mm penetration distance after the first 150 mm. The blow counts are then corrected for the energy transferred to the rod head, so that the number corresponds to a 60% energy transfer ratio. Figure 9 shows the corrected blow counts at each depth and demonstrates that the BH1 and BH2 profiles at site A are similar. Compared with site A, the equivalent N_60_ obtained at site B rapidly increases with an increase in depth. At the depth of 9 m, the equivalent N_60_ reaches 111 blows. Evidently, the equivalent N_60_ at the depths of 10 and 11 m is greater than 150 blows, which are regarded as refusal and eventually removed from the profile. Due to the difference between an SPT split-spoon sampler and the cone used in this study, the numbers should not be expected to be perfect equivalents; however, as Figure 9 demonstrates, the profiles of the equivalent N_60_ track the N_60_ obtained from the SPTs reasonably well.

**Figure 10:**
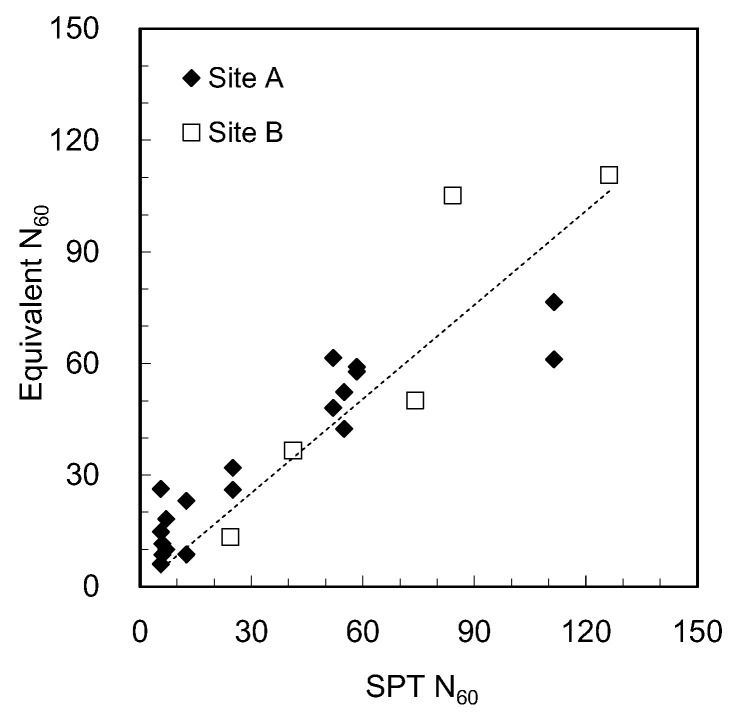
Equivalent N_60_ versus SPT N_60_.

The relationship between N_60_ and the equivalent N_60_ obtained from sites A and B is plotted in Figure 10 after matching the measured depths for the SPT and ICP test. The slope of the linear relationship and the determination coefficient (R^2^) corresponded to 0.84 and 0.79, respectively. Similarly to the profile of the equivalent N_60_, the data at the depths of 10 and 11 m at site B, which were regarded as refusal, were excluded from the relationship. In fact, the dynamic cone penetration tests using the ICP were often conducted within a 450 mm penetration distance, and the subsoil within a 450 mm penetration distance might be weaker than that at the penetration depth of 450 mm. Accordingly, the equivalent N_60_ was smaller than the N_60_.

## 4. Dynamic Response Analysis

**Figure 11:**
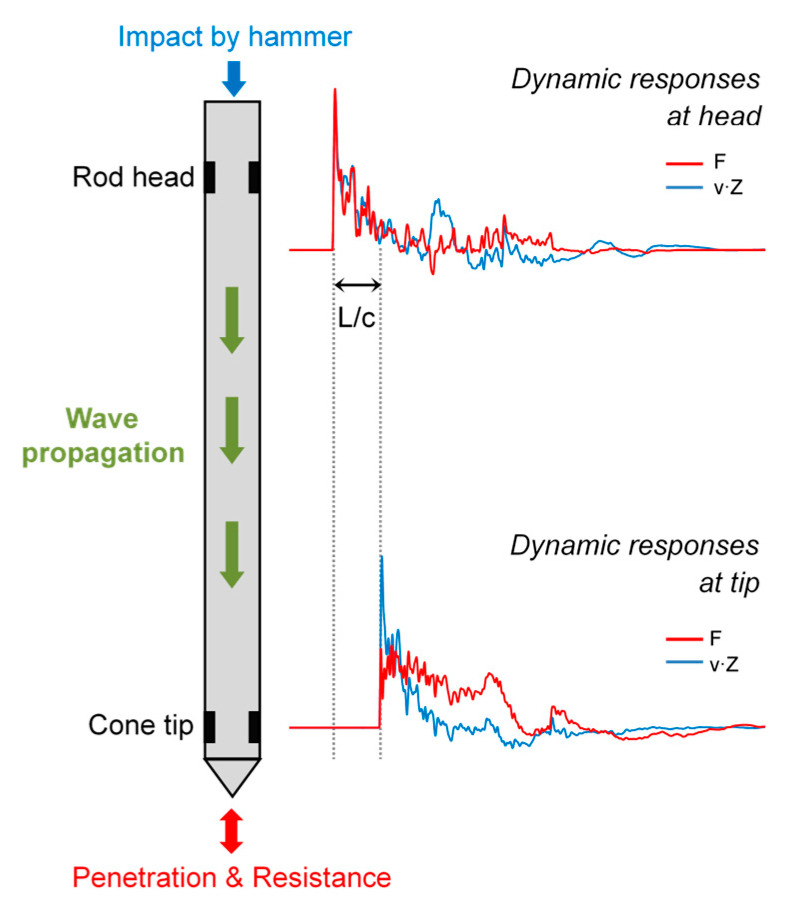
Schematic diagram of the force and velocity waveforms measured from the ICP. L—the total length of the rods at each depth, c—the wave velocity through steel.

In the dynamic cone penetration tests, the dynamic responses of the ICP were monitored using the accelerometers and strain gauges mounted on the cone tip and rod head (Figure 11). Figure 11 shows the typical force and velocity waveforms detected at both positions. As the compressional wave generated by the applied energy travels down to the cone tip, the force and velocity waveforms were detected earlier at the rod head than at the cone tip. The lag between the two is, as expected, L/c, where L denotes the total length of the rods at each depth, and c denotes the wave velocity through steel.

### 4.1. Instrumentation Verification

**Figure 12:**
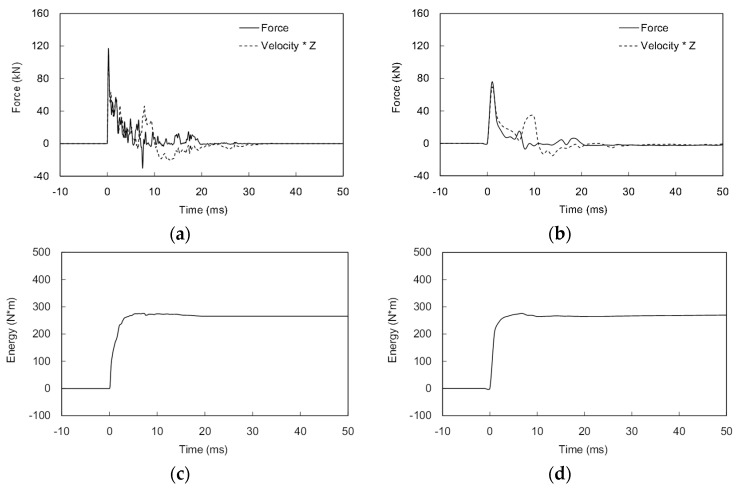
Comparison of the dynamic measurements: (**a**) force and velocity at the ICP rod head; (**b**) force and velocity at the PDA sensors; (**c**) energy at the ICP rod head; (**d**) energy at the PDA sensors.

The measurements obtained from the instrumentation at the rod head were compared with those from the PDA sensors. Figure 12a,b shows the typical force–velocity signals obtained from the PDA and ICP. Due to the difference in sampling frequency, the first peak values of the force and velocity obtained from the ICP at a sampling rate of 96 kHz are greater than those obtained from PDA at a sampling rate of 1 kHz. However, the trends of the force and velocity obtained from both the ICP and PDA are similar. Furthermore, the estimated maximum transferred energy at the rod head of the ICP is essentially the same for the ICP sensors and PDA (Figure 12c,d). Thus, an equally good performance can be expected because the accelerometer and strain gauges installed on the cone tip are identical to those installed on the rod head.

### 4.2. Force and Velocity

**Figure 13:**
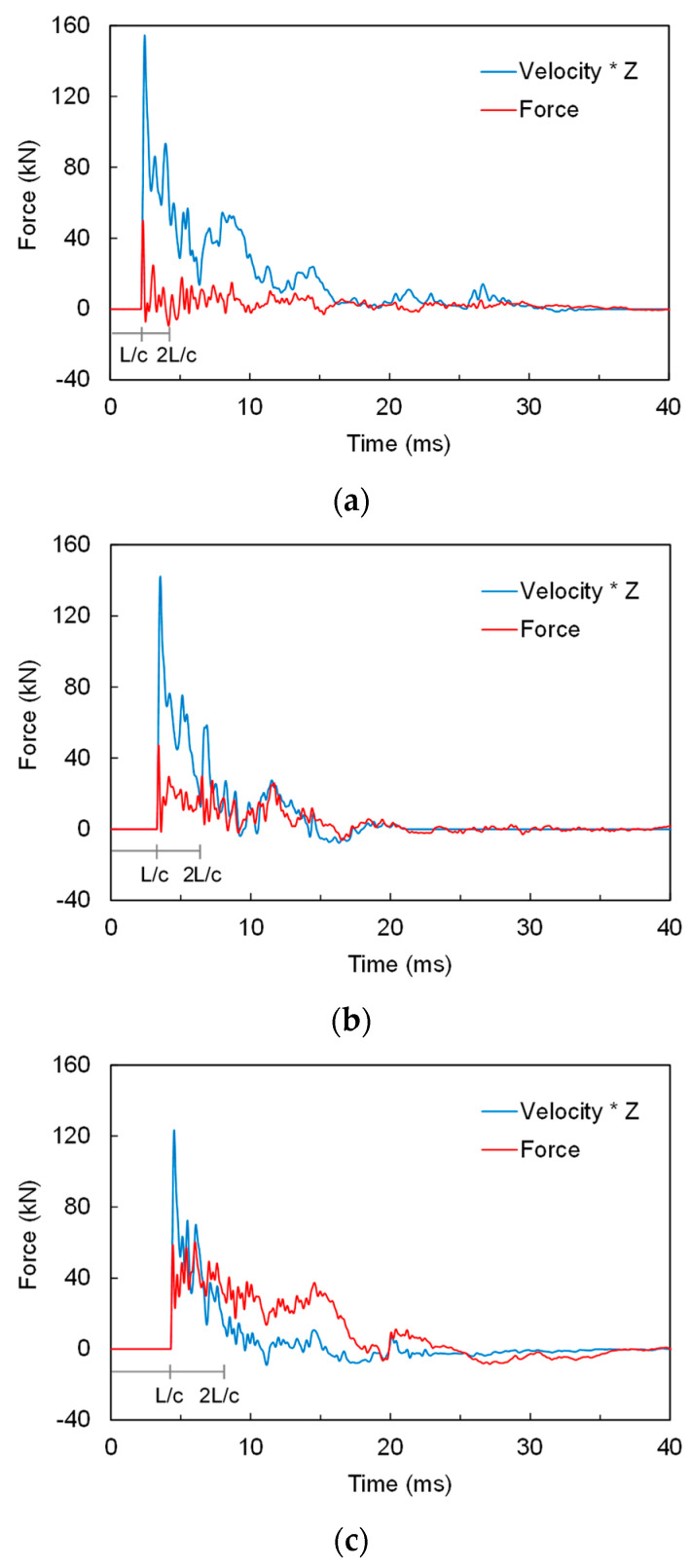
Typical dynamic responses measured at the penetration depth of 100 mm: (**a**) 8 m; (**b**) 14.5 m; (**c**) 20 m. Zero on the x-axis corresponds to the initial rising time of the force and velocity at the rod head.

Based on the one-dimensional wave mechanics, the force and velocity measured at the end are influenced by the boundary condition of the end, where the superposition of incident and reflective waves occurs. Figure 13 shows the typical dynamic response detected at the cone tip at three depths: 8, 14.5, and 20 m. The force and velocity signals measured at the cone tip are plotted in Figure 13a. At a depth of 8 m, the magnitude of the peak force is small, whereas the peak velocity is large and nonzero velocity is sustained over a relatively long time. Conversely, at a depth of 20 m, the peak force is large, and the nonzero force is sustained over a relatively long time, whereas the amplitude and duration of the velocity signal are reduced. The magnitudes and durations of the force and velocity signals in the dynamic cone penetration test are primarily a function of the resistance experienced by the cone tip [14]. The initial rising time detected at the rod head was set to zero, and that detected at the cone tip was L/c. As the rod length increases with increasing depth, the time corresponding to L/c also increases.

### 4.3. Total Cone Resistance

The soil resistance was estimated from the dynamic penetration tests [34,35,36,37]. Schnaid et al. [34] suggested an SPT dynamic force, considering a sampler energy and penetration distance; according to their approach, calibration of several coefficients is required to estimate the dynamic force. Kianirad [37] used the maximum-likelihood estimation method to model the soil resistance by the dynamic response of the cone tip force. However, the rising and falling times of an optimized square pulse should be defined by the operator in the force–time history obtained from the strain gauge. In this study, the measured dynamic response of the cone tip force was used as a soil resistance index.

Total cone resistance (q_t_), including both the static and dynamic components, can be estimated by dividing the cone tip force integrated within a certain time range by the elapsed time (∆t) and the cross-sectional area (A) of the cone tip:

(2) $$q_{t}=\frac{1}{A}\frac{\int_{L/c}^{2L/c}F_{\mathrm{tip}}\left(t\right)\mathrm{dt}}{\Delta t}$$

where F_tip_ denotes the force detected at the cone tip using the strain gauges; ∆t is the time elapsed from L/c to 2L/c, which is equal to L/c. L/c and 2L/c indicate the times elapsed from the initial rising time detected at the rod head (Figure 13). The lower integration limit (L/c) corresponds to the time at which the penetration process starts at the cone tip. The upper integration limit (2L/c) was chosen because, within the time range (L/c to 2L/c), a substantial penetration exceeding the conventional values of “soil quake” would have been achieved; this allows the consideration of whatever additional action occurs after L/c without effects of a second-cycle compressional wave, which would cause additional penetration [38]. Note that the total cone resistance represents an average stress within a specific time range obtained at the cone tip.

**Figure 14:**
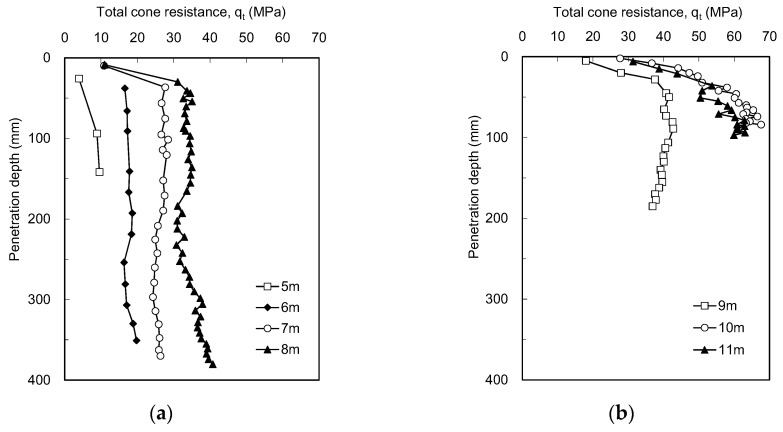
Profiles of the total cone resistance obtained at each depth at site B: (**a**) 5–8 m; (**b**) 9–11 m. Note that the data points at all depths indicate the penetration depths at every three blows.

The profiles of the total cone resistance estimated at each depth at site B are plotted in Figure 14. At the depths of 5–7 m, the q_t_ was obtained from the dynamic cone penetration tests following the static cone penetration tests, whereas the dynamic cone penetration tests were conducted only at the depths of 8–11 m. For the depths of 5–8 m, the q_t_ ranges from 4 to 40.7 MPa, and the resistance remains almost constant along the penetration depth. Conversely, the q_t_ at the depths of 9–11 m increases with increasing penetration depth. At a depth of 9 m, the value of q_t_ reaches 42.7 MPa and then slightly decreases. The q_t_ at the depths of 10 and 11 m reaches 67.6 and 63.1 MPa, respectively.

**Figure 15:**
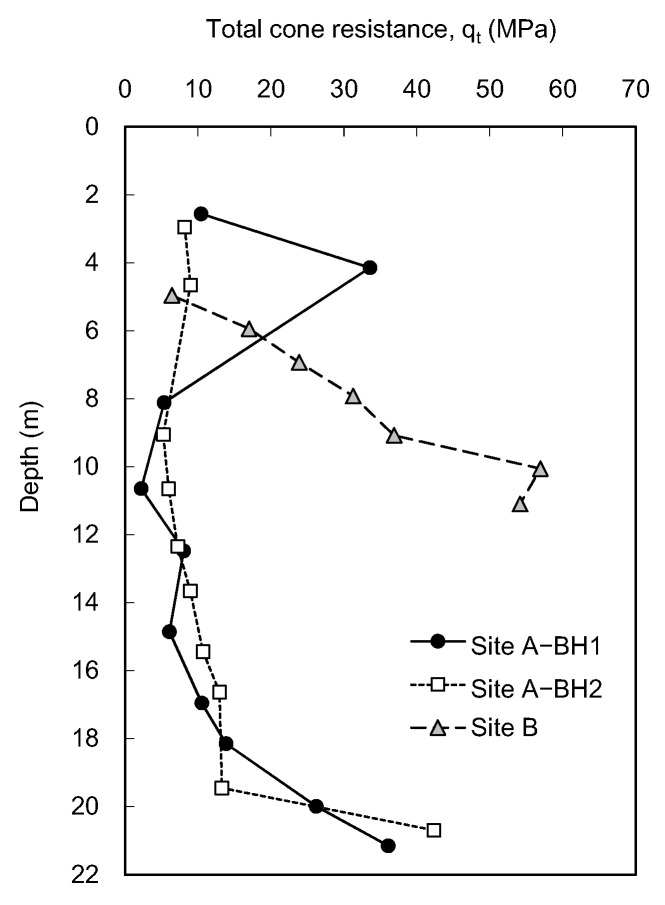
Profiles of the average total cone resistance.

The total cone resistance averaged for the first 90 mm penetration obtained at sites A and B is plotted in Figure 15. The trends of q_t_ along the depth are almost identical between BH1 and BH2, except for the upper layer at site A, which consists of large particles. The q_t_ at site B rapidly increases with the depth compared with site A.

## 5. Correlations with Total Cone Resistance

### 5.1. Total Cone Resistance Versus N Value

**Figure 16:**
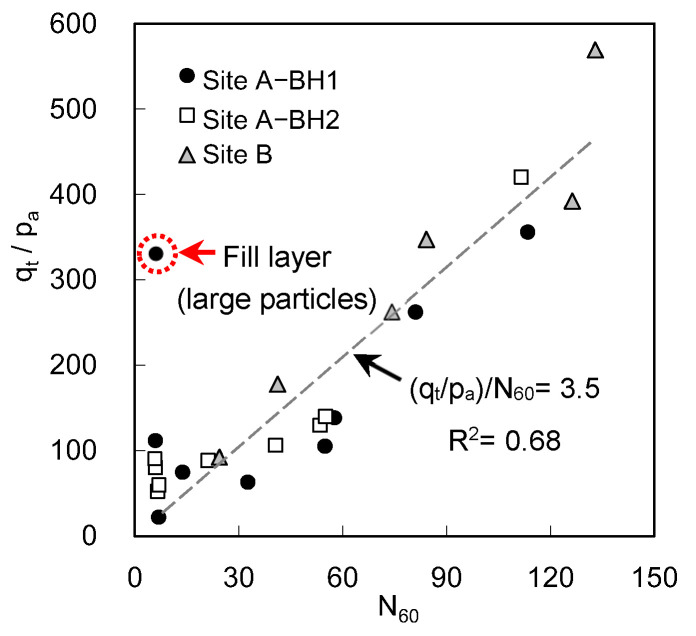
Comparison of the average total cone resistance obtained at each depth using ICP with N value measured from SPT. The p_a_ denotes the reference stress of 100 kPa.

To demonstrate the correlation between the total cone resistance and the corrected blow count, the N_60_ and average q_t_ at the same depths of sites A and B are plotted in Figure 16. The ratio of (q_t_/p_a_)/ N_60_ which resulted from two SPTs and three dynamic cone penetration tests is set to 3.5. Robertson et al. [9] demonstrated the variation of (q_c_/p_a_)/N along the median diameter D_50_ of the subsoil in SPT and CPT. Considering that the D_50_ at site A ranges from 0.13 to 0.58, except for the constructed fill layers corresponding to the 2 and 4 m depths, the value of (q_t_/p_a_)/N_60_ obtained in this study is slightly underestimated compared with the general trend of (q_c_/p_a_)/N reported by Robertson et al. [9]. Based on an improved correlation by Kulhawy and Mayne [39], the (q_t_/p_a_)/N_60_ values predicted for the fill layers correspond to 6.2 and 7.9, and those predicted for other depths range from 3 to 4.7, which are in the range of the dataset provided by Kulhawy and Mayne [39].

### 5.2. Total Cone Resistance Versus Dynamic Cone Penetration Index

**Figure 17:**
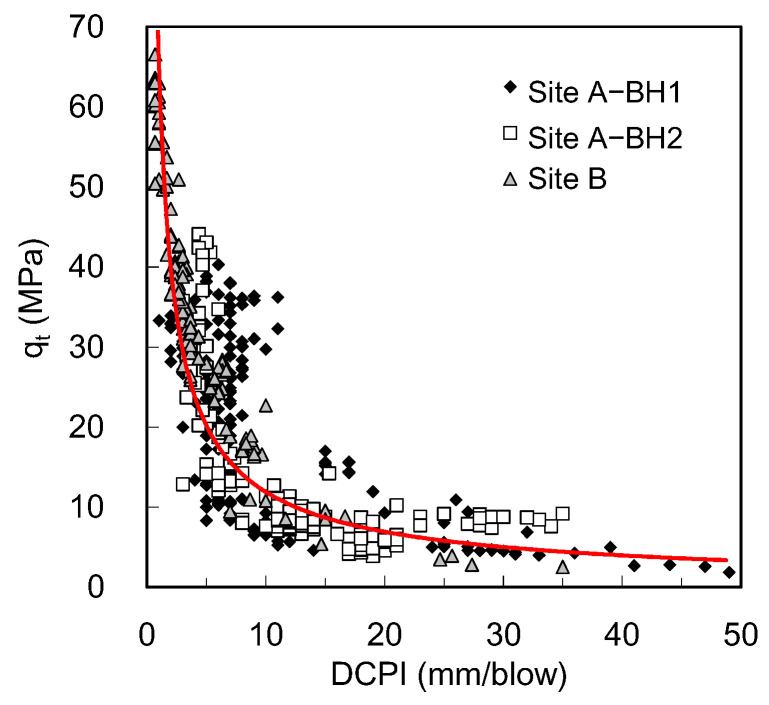
Correlation between the total cone resistance and dynamic cone penetration index. The red line indicates the trend line for the entire data set.

The soil resistance in the dynamic cone penetration test is commonly represented as a dynamic cone penetration index (DCPI), which indicates the penetration distance per blow. For comparison, the q_t_ and DCPI obtained at every single blow are plotted in Figure 17. The correlation between the q_t_ and DCPI is expressed as follows:

(3) $$q_{t}=a\cdot {\mathrm{DCPI}}^{b}$$

where a and b are constants determined by regression analysis; the values of a and b are summarized in Table 2. The q_t_ increases with decreasing DCPI. Because the subsoils of site A are deposited as a multilayer, the DCPI at site A exhibits a wide range, from 2 to 49 mm/blow. Most data points at site B are concentrated under the DCPI of 10 mm/blow. The DCPI data at site B shows a higher coefficient of determination compared with site A (Table 2). It was found that the q_t_ can represent soil resistance with high resolution more than the DCPI based on the strong correlation at high soil resistance. Especially in the dense layer, where the driving of cone is difficult or refused, the estimation of q_t_ under a few blows can be effectively used as an alternative to the DCPI determined from a number of blows.

### 5.3. Total Cone Resistance Versus Static Cone Resistance

**Figure 18:**
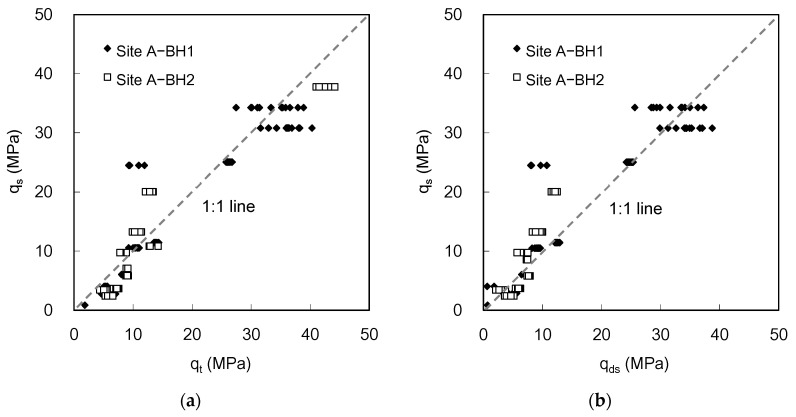
Comparison of the static cone resistance obtained using ICP—q_s_ with (**a**) the total cone resistance—q_t_ and (**b**) static component of the total cone resistance—q_ds_.

The static and dynamic cone penetration tests performed at each depth in a borehole produced the static cone and total cone resistances. At each depth, the average static cone resistances obtained for the 45 mm penetration prior to dynamic penetration were compared with the total cone resistance obtained for the 90 mm penetration after the completion of the static penetration. The relation between q_t_ and q_s_ at site A is plotted in Figure 18a and demonstrated as follows:

(4) $$q_{s}=\alpha \cdot q_{t}\;\;\;(R^{2}\;=\;0.88)$$

where α is a constant and the value of α is 0.94. Note that the effect of borehole-drilling operations on q_s_ remains at several depths. The results indicate that the total cone resistance estimated from the dynamic response is generally greater than the static cone resistance due to the damping effect.

To evaluate the damping effect on the total cone resistance, a damping coefficient (C_Lysm_) proposed by Lysmer and Richart [40] was employed as follows:

(5) $$C_{\mathrm{Lysm}}=\frac{3.4R^{2}}{1-\upsilon }\sqrt{\rho G_{max}}=\frac{3.4R^{2}}{1-\upsilon }\rho V_{s,max}$$

where R denotes the radius of the cone penetrometer; υ is Poisson’s ratio of the soil; and ρ is the mass density of the soil. As proposed by Baldi et al. [41] and Mayne and Rix [42], the shear wave velocities V_s_ were estimated from the correlations between V_s_ and q_s_ to evaluate the damping coefficient C_Lysm_. At each depth, the velocities measured at the cone tip were averaged over the duration between L/c and 2L/c. As presented in Table 3, the dynamic cone resistance (q_d_) was obtained by multiplying the damping coefficient C_Lysm_ and the average velocity of the cone tip. The dynamic cone resistance estimated in clay is greater than that estimated in sand.

The total cone resistance was corrected to a static component of the total cone resistance q_ds_ (=q_t_ − q_d_) to exclude the effect of the dynamic cone resistance. The relation between q_ds_ and q_s_ is plotted in Figure 18b. The constant α increases to 1.01 with the determination coefficient (R^2^) of 0.85, which indicates that the q_ds_ is almost identical to the q_s_. For correction, data at depths deeper than 17 m in BH2, where the velocity at the cone tip was not obtained, were excluded. Considering that the q_t_ averaged along the 90 mm penetration distance is similar to the q_s_ within the wide range, the results imply that the q_ds_ can be used as an alternative to q_s_, and the uncorrected q_t_, including the damping effect, should be used with caution for the estimation of q_s_ in clay. 

## 6. Summary and Conclusions

In this study, the ICP, which can detect the dynamic responses at the cone tip, was employed for dense layer characterization and soil resistance estimation. Using the dynamic response of the cone tip of the ICP, the total cone resistance was proposed as a soil resistance index. The ICP, including the cone tip and rod head, was designed to detect the dynamic responses of the subsoil. The dynamic responses obtained from the ICP and PDA instruments were compared at the rod head to verify the performance of the accelerometer and strain gauges installed on the ICP. The ICP and PDA instruments provided not only similar trends of force and velocity but also identical values of the maximum transferred energy.

For the application of the ICP, the static and dynamic cone penetration tests were conducted in three boreholes at two sites. At each depth, the dynamic cone penetration tests were conducted after the completion of the static cone penetration tests. The results revealed that the average static cone resistance and equivalent N_60_ in two boreholes at site A were almost identical and that the increase in the average static cone resistance and equivalent N_60_ along the depth at site B was faster than that at site A. In the entire depth, the profiles of the average static cone resistance and equivalent N_60_ obtained from the ICP were similar to that of N_60_ obtained from the SPT. In addition, the soil properties sampled at site A demonstrated that the subsurface ground is multilayered with different soils.

The analysis of dynamic responses at the cone tip started from the force and velocity detected at the cone tip. Based on the dynamic response of the cone tip force, the total cone resistance was defined as the ratio of the cone tip force integrated within a certain time range to the elapsed time and the cross-sectional area of the cone tip. In the dynamic cone penetration tests, the total cone resistances were estimated at each blow. The total cone resistance is strongly correlated with the three soil resistance indices compared with the results of the SPTs, dynamic cone penetration indices, and static cone resistances. Specifically, the total cone resistance was used effectively for dense layer characterization, where the low DCPI and high static cone resistance occurred. Therefore, the total cone resistances would be acceptable for the static cone resistance in the dense layers where the CPT cannot be performed due to the lack of reaction force. The novel approach for soil resistance estimation using the ICP may be a useful method for a prompt and quantitative characterization of the dense layers.

## Figures and Tables

**Table 1:** Properties of soil sampled at site A.

Depth (m)	N_60_	Sampling Depth (m)	Specific Gravity G_s_	Median Diameter D_50_ (mm)	Gradation Coefficient C_c_	Uniformity Coefficient C_u_	Water Content w_c_ (%)	Liquid Limit LL (%)	Plastic Limit PL (%)	Plasticity Index PI (%)	US CS *
0–3.3	6	2.3	2.88	1.64	0.31	26.76	14	-	-	-	SP
3.3–5.3	6	4.3	2.72	4.12	0.94	38.3	16	-	-	-	SP
5.3–7.3	2	6.3	2.68	0.13	0.71	2.54	37	31	27	4	ML
7.3–9.3	7	8.3	2.72	0.49	0.77	3.43	26	45	23	22	CL
9.3–11.3	6	10.3	2.71	0.34	0.59	3.12	32	39	28	11	CL
11.3–13.3	13	12.3	2.72	0.33	0.7	2.61	32	34	29	5	SM
13.3–15.3	25	14.3	2.66	0.42	0.84	3.08	24	33	27	6	SM
15.3–17.1	52	16.3	2.68	0.49	0.8	3.52	28	33	27	6	SM
17.1–18.6	58	17.8	2.65	0.58	0.9	3.54	18	32	26	6	SM
18.6–20.1	55	19.3	2.59	0.5	0.86	3.09	24	32	28	4	SM
20.1–21.6	112	20.8	2.66	0.56	0.85	3.07	14	26	23	3	SM

* USCS denotes unified soil classification system.

**Table 2:** Parameters of the correlation between the total cone resistance and dynamic cone penetration index.

	a	b	R^2^
Site A	72.33	−0.773	0.60
Site B	65.23	−0.641	0.82
Entire	70.87	−0.757	0.73

**Table 3:** Estimation of the dynamic cone resistance based on Lysmer’s analog.

Depth (m)	Shear Wave Velocity V_s_ (m/s)		Damping Coefficient C_Lysm_ (kN·s/m)		Average Velocity at Cone Tip V_tip_ (m/s)		Average Dynamic Cone Resistance q_d_ (MPa)	
	BH1	BH2	BH1	BH2	BH1	BH2	BH1	BH2
0–3.3	120.1	117.0	0.53	0.52	3.73	5.10	1.27	1.69
3.3–5.3	192.8	162.4	0.95	0.80	2.69	3.14	1.63	1.61
7.3–8.3	516.4	233.2	2.30	1.04	3.21	-	4.76	-
8.3–9.3	292.8	280.2	1.31	1.25	-	2.93	-	2.36
10.3–11.3	199.7	241.1	0.89	1.08	3.30	2.42	1.89	1.67
12.3–13.3	206.8	200.1	0.91	0.88	2.70	2.30	1.59	1.31
13.3–14.3	211.4	215.1	0.93	0.95	-	2.18	-	1.33
14.3–15.3	217.3	233.7	0.96	1.03	2.38	-	1.47	-
15.3–16.1	233.6	248.4	1.06	1.13	-	2.11	-	1.53
16.1–17.1	247.2	262.4	1.15	1.22	1.87	1.22	1.38	0.96
17.1–18.6	263.4	260.0	1.23	1.21	1.80	-	1.42	-
18.6–20.1	280.2	258.7	1.34	1.24	1.71	-	1.47	-

## Notations

BC_100_	()	Cumulative blow count numbers corresponding to the first 100 mm penetration
c	(m/s)	Wave velocity through steel
C_Lysm_	(kN·s/m)	Damping coefficient
C_R_	()	Factor of rod length
D_50_	(mm)	Median diameter
DCPI	(mm)	Dynamic cone penetration index
ER	(%)	Energy ratio
F_tip_	(kN)	Force detected at the cone tip
L	(m)	Total length of the rods at each depth
N value	()	Blow count measured in SPT
N_60_	()	N value corrected by the effects of the rod length and transferred energy
p_a_	(kPa)	Reference stress of 100 kPa
q_c_	(MPa)	Cone tip resistance measured by CPT
q_d_	(MPa)	Dynamic cone resistance estimated from the dynamic cone penetration tests of ICP
q_ds_	(MPa)	Static component of the total cone resistance (= q_t_ − q_d_) in dynamic cone penetration tests
q_s_	(MPa)	Static cone resistance measured from the static cone penetration tests of ICP
q_t_	(MPa)	Total cone resistance estimated from the dynamic cone penetration tests of ICP
R	(m)	Radius of the cone penetrometer
V_s_	(m/s)	Shear wave velocities
V_tip_	(m/s)	Average velocity measured at the cone tip of the ICP
ρ	(kg/m^3^)	Mass density of the soil
υ	()	Poisson’s ratio of the soil
